# Development of lower limb training interventions that promote an external focus of attention in people with stroke: a modified Delphi survey

**DOI:** 10.1080/09593985.2021.1972501

**Published:** 2021-09-03

**Authors:** Louise Johnson, Jane Burridge, Sara Demain

**Affiliations:** a Stroke Service, University Hospitals Dorset NHS Foundation Trust - Stroke Service, Royal Bournemouth Hospital Castle Lane East Bournemouth Dorset, Bournemouth, UK; b School of Health Sciences, Faculty of Environmental and Life Sciences, Building 45, University of Southampton, Southampton, UK

**Keywords:** Stroke, rehabilitation, hemiplegia, attention, movement, gait, Delphi technique

## Abstract

**Objective:**

To produce guidance and validated examples of tasks that promote an external focus of attention, for use in lower limb rehabilitation in an inpatient stroke care setting.

**Design:**

Electronic survey, using e-Delphi methodology.

**Participants:**

A multi-professional expert panel of 14 clinicians and researchers, with expertise in stroke rehabilitation and/or motor learning.

**Method:**

Each survey round consisted of two parts: 1) classification of specific exercise examples, shown using video and 2) the categorization of specific tratement adjuncts. The panel was asked to comment on: likely focus of attention of the performer; instructions that would promote an external focus of attention; and how the task set-up could be modified to promote an external focus of attention. Rounds 2 and 3 included a summary of results from the previous round, and revised/new examples. The panel were also asked to state their level of agreement with a series of statements that arose from the free text. Three rounds of survey were completed and the a priori threshold for agreement was set at 80%.

**Results:**

Eighteen iterations of exercises were presented, and 12 were accepted as promoting an external focus of attention. In addition, six additional statements were generated based on open responses, leading to further specific guidance on facilitating an external focus of attention in clinical practice.

**Conclusions:**

Commonly used rehabilitation exercises can be adapted to promote an external focus of attention, by altering the therapist’s use of instructions and/or altering the task set up. Treatment principles and examples of tasks that promote an external focus have been produced.

## Introduction

The important role of instructional statements is widely acknowledged in the motor-learning literature. Their nature, timing, frequency, and type can impact both performance and learning (Maier, Ballester, and Verschure, 2019; Wulf, 2013). One mechanism through which verbal instructions influence learning, is the way in which they direct focus of attention (Wulf, Höß, and Prinz, 1998). This can be either internal where the learner is focusing on body movements or external where the learner is focusing on the task outcome or the environment. Research in healthy populations has shown the relative benefits of adopting an external focus of attention during skill acquisition (Wulf, 2013). This finding that has been replicated across a wide range of tasks as varied as: long jump (Porter, Ostrowski, Nolan, and Wu, 2010); golf (Wulf and Su, 2007); and postural sway (Richer, Saunders, Polskaia, and Lajoie, 2017). These benefits are thought to result from reducing attentional demand (Liao and Masters, 2001; Maxwell, Masters, and Eves, 2003); limiting the accrual of declarative knowledge (Maxwell, Masters, and Eves, 2000); and enabling movement to be governed automatically (Wulf, McNevin, and Shea, 2001; Wulf and Prinz, 2001).

The findings from studies involving people with stroke are less consistent. External focus conditions have led to improved performance in lateral weight transfer in sitting (Muckel and Mehrholz, 2014) and reach to grasp (Durham, Van Vliet, Badger, and Sackley, 2009; Fasoli, Trombly, Tickle-Degnen, and Verfaellie, 2002). However, other studies found no benefits of an external focus of attention when people with chronic stroke practiced: reaching (using robotics) (Kim et al., 2017); seated stepping (Kal et al., 2015); balance (using a balance platform device) (Kal et al., 2019); or gait tasks (Jie et al., 2018).

Typically, studies in this field use controlled experimental designs. They evaluate a defined and often novel activity, with a select patient group. Differences in the type of task, stage of learning, and clinical characteristics of the participants (including chronicity and participant preference) may all influence the relative benefits of each strategy, and make comparisons across studies challenging. Studies also vary in the method used to generate an external focus, with some trials using scripted instructional statements (Durham et al., 2014; Fasoli, Trombly, Tickle-Degnen, and Verfaellie, 2002; Muckel and Mehrholz, 2014), and others generating an external focus through the use of analogies (Jie et al., 2018), or dual tasks (Kal et al., 2015). While this standardization of approach gives methodological robustness within trials, and is invaluable in the early stages of testing theory, it can limit translation into clinical settings, where the approach must be adapted for the individual patient.

While current evidence does not convincingly demonstrate superiority of either an internal or external focus of attention in stroke rehabilitation, the concept could be especially important given that: 1) rehabilitation goals typically relate to the recovery of previously learned (autonomous) movements, such as reach to grasp, sit to stand and walking; and 2) impairments of cognition and language, alongside factors such fatigue, may limit an individual’s capacity for learning environments that require high levels of information processing. Thus, tailored approaches may be required (Kal et al., 2019), and further work is required to understand how best to apply different learning models in practice, what works best, and for whom.

Before we can investigate the role that focus of attention may have on the motor recovery of people with stroke in the clinical setting, we first need to define how rehabilitation activities can be adapted to encourage the performer to direct their attention externally. This is important as observational studies have highlighted that physiotherapists use frequent verbal instructions (Johnson, Burridge, and Demain, 2013; Stanton, Ada, Dean, and Preston, 2015; Talvitie, 2000) and tend to favor an internal focus of attention (Johnson, Burridge, and Demain, 2013).

In this paper, we describe the use of a modified Delphi approach, to develop guidance for training mobility using an external focus of attention. The Delphi technique is a method of gaining consensus among a panel of experts (Brady, 2015). It enables the rapid and efficient collection of opinions, through a series of questionnaires, interspersed with controlled feedback (Rowe and Wright, 1999). Questions are refined, based on previous feedback, until there is convergence of opinion (Hsu and Sandford). As this method enables a group of people with diverse experience to contribute to the sharing and refinement of ideas, it is valuable when developing interventional guidance (Brady, 2015). The aim of this study was to define and describe how rehabilitation activities can be adapted to promote an external focus. The study concentrates on exercises targeting improvements in sit to stand, stepping/transfers and gait. Our intention was to produce clinically focused guidance, supported by a suite of examples, to demonstrate the concept in practice. This forms the first stage in a programme of research, which is examining the benefits of an implicit learning approach for recovery of lower limb function, in early stroke rehabilitation. Directing attention externally is a key component of this approach.

## Methods

### Study Design

Ethical approval was granted from the University of Southampton Ethics and Research Governance Committee (ERGO), Ref: 42008. This study used a modified e-Delphi method. Unlike traditional Delphi methods, where the initial round is open to allow panel members the freedom to express their views (Trevelyan and Robinson, 2015), we started with a series of focused questions. This approach is deemed appropriate if basic information concerning the target issue is available and usable (Hsu and Sandford, 2007), as it avoids the generation of unmanageable quantities of data (Trevelyan and Robinson, 2015). Previous researchers have used Delphi methodology to report consensus relating to the broad behaviors that contribute to learning environments being implicit, of which directing attention externally is a principal component (Kleynen et al., 2020, 2014). Our aim was to reach agreement on how *specific* exercises can be delivered, while promoting an external focus; thus informing the content of the treatment guidance that we will later use in a clinical trial. Starting with focused questions relating to exercise examples enabled a proportionate amount of data to be generated.

### Sampling and Recruitment

In line with recommendations (Hsu and Sandford, 2007), we aimed to recruit a panel of between 8 and 12 people who had experience and knowledge in the field of motor learning and/or stroke rehabilitation. Specific criteria were: hold a related professional qualification; at least 5 years’ experience in a related field of work; has an understanding of the concept of focus of attention in motor learning; evidence of post-graduate professional development in the field of motor learning (e.g., further professional study and involvement in research); willing and able to commit to 3–4 survey rounds. Through non-probability sampling, we aimed to establish a diverse but appropriate panel, to include both clinicians and researchers from a range of backgrounds and geographical locations not limited to the UK.

Panelists were identified through the professional networks of the research team, and through knowledge of those working in the field of interest (e.g., academics with published research in this field). Pre-defined response targets were set. To ensure a range of views, we aimed for a minimum of 10 participants in Round 1. Our target was to retain 80% of participants in subsequent rounds.

Potential participants were contacted via e-mail, outlining the purpose of the research, inclusion criteria, and what involvement would consist of. They were given opportunity to opt out either by replying to the researcher or by non-completion of the survey. Prior to inclusion in the study, panelists were asked to confirm that they met the inclusion criteria.

## Procedures

### Developing the questionnaire

An online survey tool (SurveyMonkey®) was used for data collection. The tool was initially piloted with two clinical-academic physiotherapists, who were not otherwise involved in the study. Minor changes to the format and wording were made as a result of the pilot feedback.

A link to the survey was circulated via e-mail, approximately one week after the first contact. The opening page acted as the Participant Information Form, with implied consent through voluntary participation. Participants were given approximately three weeks to complete each round, with a reminder sent after the second week.

The survey consisted of two parts: 1) the classification of specific exercise examples, according to their focus of attention; and 2) the categorization of specific treatment adjuncts, according to their role in directing focus of attention. Panelists were asked to base their answers on the following definitions: 1) External Focus of Attention: when the performers attention is directed to the effect of the action; and 2) Internal Focus of Attention: when the performers attention is directed to the action itself (Wulf, 2007).

**Figure 1.**
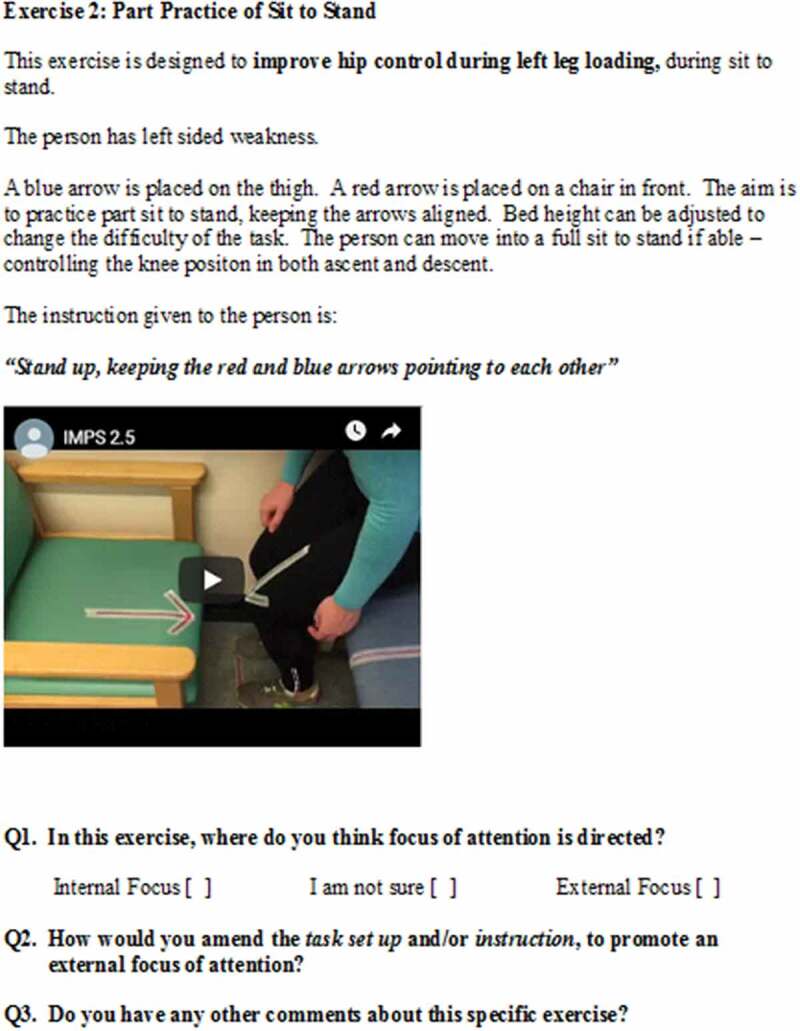
Example question, with short video demonstrating the exercise. Questions 2 and 3 were only asked if the panelist responded “internal focus” or “I am not sure” to Question 1.

Panelists were shown a series of videos demonstrating training exercises, which were developed by the primary researcher (LJ). Each video was accompanied by a written description of the task, and the given instructional statement. Panelists were asked to state where they thought the performers’ attention would be directed, with three response options: internal focus, I am not sure, external focus. If they answered internal focus or unsure, they were asked how they would modify the instruction and/or the task set-up, to bias an external focus of attention. They were also given opportunity to provide any other general feedback relating to each exercise, with an open text box. An example question is shown in Figure 1.

### Survey rounds

**Figure 2.**
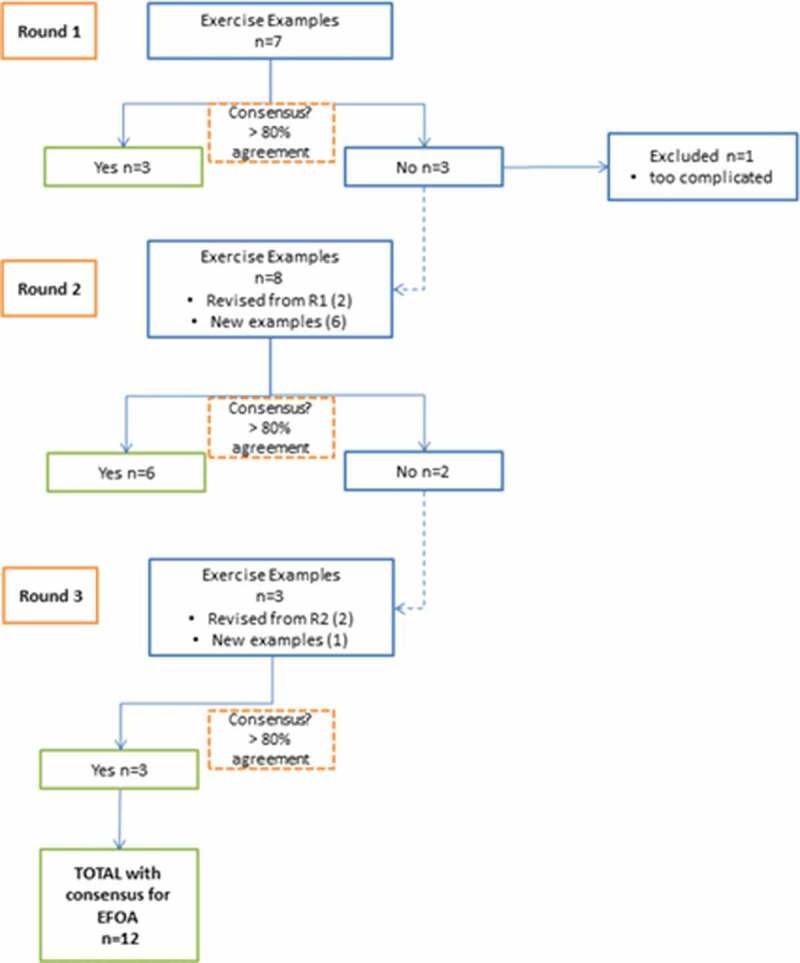
Flow chart for survey rounds and consensus.

Figure 2 outlines the survey rounds. The exercises in Round 1 were based on definitions of focus of attention given in the literature, and knowledge of exercises that could potentially be used in stroke rehabilitation. The survey included a range of examples that could feasibly promote either an internal or external focus of attention, as well as some that were purposefully ambiguous. This enabled the expert panel to generate new ideas and contribute to the next set of exercises through an iterative process, rather than simply validate an existing set of exercises that were already refined.

The questionnaire in Rounds 2 and 3 included a summary of the results from the previous round. Following this, two types of question were included. Firstly, revised and new exercises videos were presented, and participants were asked the same set of questions regarding focus of attention. Secondly, specific questions relating to topics raised in the previous questionnaire were included. A topic was considered for inclusion if it was clearly relevant to the aims of this study, and was either raised by more than one respondent, and/or there is ambiguity in the literature regarding its relevance in relation to focus of attention. For example, insights generated through the content analysis highlighted potentially conflicting views with regards to certain aspects of directing focus of attention in clinical practice, such as use of visual feedback. Where this occurred, a specific question was included in the subsequent survey round to seek wider opinion, gain clarity, and where possible, agreement from the panel.

### Data analysis

Data were analyzed using descriptive (quantitative) and content (qualitative) analyses. Response rates and levels of agreement were described using percentages. The content of open-ended data was analyzed, to identify concepts relevant to the research question. Both the descriptive and the content analyses were then considered together, in order to decide whether to accept, reject, or amend the proposed exercise. Therefore, to be included in the final treatment guidance, the exercise had to reach consensus for an external focus of attention and be viewed positively through the open-ended data. This process of analysis was completed by the primary researcher (LJ), and discussed for corroboration with a second researcher (JB).

## Results

### Participants and response rates

Twenty-three people were invited to take part in the survey. One opted out, as their immediate colleagues were participating and their views were likely to be similar. Therefore, 22 people were sent the link for each round. At the request of the ethics committee, survey completion was entirely anonymous (e-mail and Internet Protocol tracking features of the data collection tool were disabled). We were not, therefore, able to identify which participants took part in each round, and the invitation was sent to the full cohort each time.

Fourteen people responded to Round 1, however, one participant only completed their demographic details, and did not answer any survey questions. Their data were excluded, leaving 13 completed surveys for analysis. This represents an initial response rate of 59% (13/22), which met our pre-determined minimum target of 10 participants. Response rates were 64% (14/22) and 55% (12/22) in Rounds 2 and 3, respectively. The actual group of panelists differed slightly in each round (Table 1), which was a consequence of the ethical requirement for complete anonymity. The overall attrition rate was low, with a net loss of one person between Round 1 and Round 3 (1/13; 7.7%). Participants were from a mix of clinical and academic backgrounds, and from a range of professions, as outlined in Table 1.

**Table 1.** Professional background of panelists for each survey round.

Participant #	Profession	Area of Work
**Round 1**		
1–1	Physiotherapist	Clinical and Research
1–2	Sport Scientist	Research and Education
1–3	Physiotherapist	Research and Education
1–4	Physiotherapist	Research and Education
1–5	Physiotherapist	Education
1–6	Physiotherapist	Research and Education
1–7	Physiotherapist	Education
1–8	Physiotherapist	Clinical and Research
1–9	Physiotherapist	Clinical
1–10	Physiotherapist	Education
1–11	Occupational Therapist	Research and Education
1–12	Physiotherapist	Clinical and Research
1–13	Human Movement Scientist	Research and Education
**Round 2**		
2–1	Physiotherapist	Education
2–2	Physiotherapist	Clinical and Research
2–3	Clinical Psychologist	Clinical
2–4	Physiotherapist	Clinical and Research
2–5	Physiotherapist	Clinical and Research
2–6	Physiotherapist	Clinical
2–7	Physiotherapist	Clinical and Research
2–8	Skipped Question	Education
2–9	Physiotherapist	Clinical
2–10	Physiotherapist	Research and Education
2–11	Occupational Therapist	Research and Education
2–12	Physiotherapist	Education
2–13	Human Movement Scientist	Research and Education
2–14	Human Movement Scientist	Research and Education
**Round 3**		
3–1	Physiotherapist	Clinical
3–2	Physiotherapist	Clinical and Research
3–3	Human Movement Scientist	Research and Education
3–4	Human Movement Scientist	Research and Education
3–5	Physiotherapist	Clinical and Research
3–6	Physiotherapist	Clinical and Education
3–7	Physiotherapist	Clinical
3–8	Physiotherapist	Research
3–9	Physiotherapist	Research and Education
3–10	Clinical Psychologist	Clinical
3–11	Physiotherapist	Education
3–12	Physiotherapist	Research and Education

Note that due to the requirement for anonymity of respondents, each panelist was assigned a new participant number for each survey round, and it was not possible to track the responses through the rounds.

### Consensus

Across the three survey rounds, 18 exercise examples were presented. Consensus regarding the likely focus of attention derived from an exercise was reached for 13 of these. However, one exercise was rejected despite reaching threshold for consensus for an external focus of attention, as there were consistently negative comments regarding its complexity and practicality. An overview of the survey rounds is given in Figure 1.

Characteristically, the accepted exercises promoted an external focus of attention through the use of clear external reference points, or parameters with precise end points. For example: a target, marker, environmental cue, or object providing an external reference point, and an instructional statement paying reference to this (e.g., “*touch the marker*”). Examples of exercises with >80% agreement regarding an external focus of attention are given in Appendix 1.

### Content analysis

Four themes relating to focus of attention emerged from the open-ended data. Table 2 shows the percentage agreement for each statement related to these themes. Common across all responses were the themes that instructional statements should be: 1) simple, including only one or two components; 2) concise, using as few word as possible; and 3) specific, in order to reliably direct attention in the desired way. Table 2 shows the percentage agreement for the presented statements, according to theme.

**Table 2.** Round 3 questions and response rates (% agreement).

Statement	Theme	% Agreement
**Statements achieving >80% agreement**		
To promote an external focus of attention, the mention of body parts should be minimized (but not eliminated), and, The task and the instruction should always focus on the outcome of the targeted movement to maintain a bias toward an external focus (e.g., tap the *marker*). The movement required to achieve an outcome should not be mentioned (e.g., *lift your* foot; *bend your* hip). When required for clarity, the body part itself can be mentioned (e.g., tap your *foot* onto the *marker*), but efforts should be made to minimize this.	Framing Instructions	93%
Providing a **demonstration** of a task or activity, alongside an externally focused instruction, can help to clarify or reinforce the desired movement pattern (while maintaining an external focus of attention).	Visual Information	100%
Focusing on **not** doing something is not as strong as focusing on **doing** something, in terms of focus of attention. For example, rather than using an instruction like “*don’t* touch the marker,” it would be preferable to set the task up differently, and say “keep touching the marker.”	Framing Instructions	92%
**Statements with a majority agreement, but sub threshold (51–79% agreement)**		
To promote an external focus of attention, there needs to be a specific external focus reference within the instruction. For example: stand up, keeping your feet behind the line.	Instructional Content	75%
To promote an external focus of attention, time or distance measures could be used. For example: see how many times you can stand up and sit down in 1 minute.	Instructional Content	58%
**Statements not achieving a majority agreement <51% agreement**		
It will help to reinforce focus of attention, if the instruction contains a direct statement about this. For example: “Step onto the marker on the block. *Focus on* the marker” is preferable to “Step onto the marker on the block”	Framing Instructions	50%
To promote an external focus of attention, an analogy can be used. For example, stand up, like you are about to greet the Queen.	Instructional Content	42%
Visual feedback, through using a mirror placed in front of the performer, would provide a source of external focus feedback	Visual Information	17%
The concept of motor learning is less relevant to isolated strengthening exercises, such as a resisted dorsiflexion exercise . Therefore, directing a specific focus of attention is unnecessary for these types of exercises.	Task Type	17%
To promote an external focus of attention, dual tasking could be used. For example, practice standing up and sitting down, while recalling a shopping list.	Instructional Content	8%

### Theme 1: Principles for framing instructions

The panel initially presented opposing views regarding the naming of body parts, as part of the instruction. For example, panelists were shown an exercise aiming to facilitate lateral weight transfer in sitting, where the patient was instructed “*touch your shoulder against the marker*.” Although 92% agreed this would encourage an external focus, several commented on the use of “*shoulder*” within the instruction.

Panelist #14 (Round 1) answered internal focus and commented:

*“The main thing [issue] is that the original instruction referred to a body part (the shoulder), which may trigger an internal focus”*

Whereas panelist #2 (Round 1) answered external focus and commented:

*“Despite the mention of the shoulder, which could be construed as an internal focus, the shoulder touching the spot is an effect (outcome) of the targeted movement”*

This is an important issue for both research and clinical practice. While it is possible to eliminate the use of body part names in studies that use scripted instructions for a defined task, this could be challenging in clinical practice. The panel agreed that a more pragmatic approach is needed, whereby the naming of body parts and/or the action, should be minimized (but not entirely eliminated), in order to effectively promote an external focus of attention.

Two further topics relating to the instructional sentence were explored. The first was the phrasing of the instruction, whether positive (an instruction to do something) or negative (an instruction not to do something). For example, “*keep touching the red marker”* is a positive instruction, whereas “*don’t touch the blue marker”* is a negative one. Within the given exercises, there were examples of both types of instruction. In Round 3, 92% (n = 11) of participants agreed that positive instructions are preferable, and that task set-up should be amended to enable this. Several panelists attributed these benefits to knowledge of results, for example:

“ … .it’s easier to measure success when touching something, rather than not touching something. By how much do you not touch it?” [Panellist #2, Round 3]

The second topic relating to the instructional sentence concerned direct reinforcement of attentional focus. Participants were asked for their views on the use of instructions that directly tell someone where to focus their attention (e.g., *“Step onto the marker on the block. Focus on the marker”*).

Responses did not reach the threshold for consensus. While 50% (n = 6) agreed that a direct statement would help to direct focus of attention, the remaining participants were spread in their replies: 25% (n = 3) were unsure; 17% (n = 2) felt that a direct statement was unhelpful; and 8% (n = 1) did not think it mattered either way. Even where participants agreed with the statement, there was an acknowledgment that less is better, in terms of the instructional content. Respondents also commented that it may depend on the patient, for example, Panelist #1 (Round 3) wrote:

“Agree, but would probably keep things to the single instruction first but if the person did not complete as required then add in the additional instruction as to where to focus attention”

Overall, the panel supported the view that reinforced attentional focus instructions could be beneficial in some circumstances. Namely, in cases where the patient is not completing the task as required after a simple instruction, and where the therapist is confident that they have sufficient capacity to process the information.

### Theme 2: instructional content

Panelists were shown an example of whole-task practice of sit to stand, where the instruction was to “*stand up and sit down*.” While this instruction does not convey an internal focus of attention (there is no information about how to move), there was debate as to whether or not this would facilitate an external focus. Two panelists made the assumption that without an internally focused prompt, the person would likely think about the action of standing, rather than the way in which they stand, and this would keep the focus external. For example, panelist 1 (Round 3) commented:

*“Could be deemed too general for a specific focus but on the whole the person is concentrating on the task and not the body parts so this would make it a more externally focused instruction”.*

However, an opposing view was that a person’s default is more likely to be an internal focus:

“usually, patients with stroke seem to rely on more conscious control of movement (and use an IF). I would expect that patients would therefore, by default, would be more likely to focus internally when no clear focus directions are given” [Panellist #4, Round 3].

When asked specifically how this type of instruction would focus attention, the respondents were divided. Half (50%) agreed that the instruction “stand up and sit down” is too vague to elicit any particular focus of attention, while 42% stated that the focus of attention derived from this type of instruction could be either internal or external, depending on the individual. There was broad agreement that instructions must be specific, in order to reliably direct attention in the desired way. The use of an external reference point, or a measure (time or distance), were highlighted as a means to do this, although neither reached the threshold for consensus (75% and 58% agreement respectively).

### Theme 3: visual information

In both Round 1 and Round 2, participants suggested the use of demonstration as a means of reinforcing the desired movement pattern. For example, rather than using the instruction “step onto the marker with your *heel*,” the therapist could say “step onto the marker like this,” demonstrating contact with the heel. All (100%; n = 12) panelists agreed with the principle of providing demonstration, alongside an external focus instruction. Panelists also highlighted the importance of tailoring the demonstration to the individual:

“it [demonstration] may communicate subtleties of the movement pattern … … assuming the demonstration is done correctly and appreciates the individual constraints of the patient” [Panellist #3, Round 3].

Several participants referred to the use of a mirror in their suggestions, with what appeared to be conflicting views. Therefore, the Round 3 survey included a specific question relating to mirror feedback, which highlighted varied views. Fifty percent of respondents (n = 6) agreed that a mirror could be a source of either internal or external focus feedback, depending on other contextual factors, such as the accompanying verbal instruction, personal preferences, self-consciousness and the individuals tendency to consciously control movements (reinvestment). Seventeen percent (n = 2) stated that it would provide a source of Eexternal focus feedback and 17% (n = 2) stated that it would provide a source of internal focus feedback. The remaining 17% (n = 2) opted for neither, stating that a mirror would not provide specific focus of attention feedback.

Overall, participants were cautious about the use of a mirror for feedback, due to the increased processing requirements. External reference points in the environment (e.g., a wall or a door frame), were generally deemed preferable to visual feedback using a mirror, particularly for perception of vertical.

### Theme 4: task type

While the majority (88%) of the presented exercises were functional in nature, some targeted specific muscle groups, such as isolated strengthening through a resisted dorsiflexion exercise. Respondents commented on the nature of this exercise, questioning whether focus of attention was relevant. For example:

“for an external focus of attention, there should be some reasonable functionality to the exercise. This exercise is too far away from that”. Panellist #8 (Round 2)

“I am struggling with the idea that isolated strengthening exercises might not need a learning approach, but should primarily be shaped according to follow training principles”. Panellist #10 (Round 2)

Therefore, the panel were asked whether they agreed that the concept of motor learning is less relevant to isolated strengthening exercises; and that directing a specific focus of attention is unnecessary for these types of exercises.

Only 17% (n = 2) agreed that directing a specific focus of attention is unnecessary for isolated strengthening exercise, with 58% disagreeing, and the remaining 25% being unsure. Therefore, the majority view was that focus of attention is relevant to isolated strengthening exercises, but consensus was not achieved.

## Discussion

This study used a modified e-Delphi approach to develop and refine guidance for the delivery of stroke rehabilitation using an external focus of attention, including specific examples for clinical application. This forms part of the initial modeling of a complex intervention, as outlined in the Medical Research Council Framework (Craig et al., 2006), and is part of a larger programme of research investigating models of implicit learning in clinical practice, of which an external focus is a key component (Kleynen et al., 2020, 2014). This study has contributed to the development of implicit learning guidance, which is now being tested in a pilot cluster randomized controlled trial, the protocol for which is published elsewhere (Johnson, Burridge, Demain, and Ewings, 2019).

The challenge of adapting practice to promote implicit forms of learning has been acknowledged (Poolton, Masters, and Maxwell, 2006). This challenge may be amplified in a complex field such as stroke rehabilitation, as therapists must adapt to the specific circumstances of the person they are working with, designing task practice to be specific and sufficiently challenging, yet enable successful and efficient performance.

In a survey of Canadian physiotherapists, only 55% could correctly answer knowledge-based questions relating to focus of attention in the context of motor learning, and only 35% reported applying this knowledge into practice (DePaul, Barker, and Nasopoulos, 2016). Having practical guidance on how to use an external focus in the stroke rehabilitation setting, may support therapists to consider and select the approach that best suits an individual scenario.

This Delphi study enabled the design of 12 lower limb rehabilitation tasks that were deemed to promote an external focus. It also generated generic principles that may enable therapists to design exercises that have both an external focus of attention and are appropriate for people with stroke. An overview of these principles is given in Table 3.

**Table 3.** Key findings – principles for promoting an external focus of attention.

INSTRUCTIONS SHOULD:
Be **Simple** (few words and few components)
Be **Specific** (to reliably direct attention in the desired way)
Be **Positive** (directing the person *to do* something)
**Avoid** the mention of body parts and/or the movement required to achieve the outcome (e.g. bend and lift)
**TASK SET UP SHOULD:**
Include a clear **external reference point** (e.g. a target, markers, or object) or **parameter** (e.g. time or distance), with **precise end points** (e.g. a target, object, time or distance) ^a^

^a^Although these statements did not reach the threshold for consensus (> 80%), they were the highest ranking items with regards to task set up (See Table 2).

The composition and content of verbal information is one way in which focus of attention can be mediated (Chow, Davids, Button, and Koh, 2008). There was strong agreement among panelists with regards to the wording of instructional statements, and how they can be framed to bias an external focus of attention. This research also identified the importance of keeping instructional statements concise by: 1) conveying the message in as few words as possible; and 2) avoiding more than one or two pieces of information at a time. This aligns with evidence involving healthy individuals, showing that the accumulation of verbal knowledge is moderated not by the volume of instruction, but by the number of rules or movement components within those instructions (Bobrownicki et al., 2015). Reducing the number of components may be particularly important following stroke, due to possible impairments of working memory.

There was also agreement that positively framed statements (e.g. *touch the red marker*) were preferential to negative ones (e.g. *don’t touch the blue marker)*. When someone is instructed not to do something (e.g., *‘don’t touch the line’*) their gaze and attention is more likely to be drawn to the thing they are trying to avoid (Bakker, Oudejans, Binsch, and Kamp, 2006). This phenomenon, known as ironic effects, is well developed within sport. For example, studies in football and golf have demonstrated the benefits of avoiding negatively worded instructions (Bakker, Oudejans, Binsch, and Kamp, 2006; De La Peña, Murray, and Janelle, 2008), and more specifically, not referring to the unwanted behavior entirely (Bakker, Oudejans, Binsch, and Kamp, 2006). Although ‘ironic effect’ has not been specifically tested in stroke research, our experts strongly agreed that positively worded instructions should be encouraged.

The specificity of instructions also requires consideration. While instructions that are vague may not infer any particular focus of attention, they may also impact performance. Conveying specific externally focused instructions can be difficult and may be particularly challenging in clinical scenarios. For example, changing a precise internally focused instruction, such as “*keep your knee straight*,” to an externally focused one, is difficult. It requires the therapist to alter the task set up, as well as the given instruction, in order to achieve the desired movement pattern. Specific learning strategies, such as analogy learning, have been used as a means of promoting implicit learning in this type of scenario (Liao and Masters, 2001), for example, “*keep straight, like a pencil*.” To maintain an external focus alongside specificity requires careful set-up and monitoring of the task, to ensure the desired movement is achieved.

### Strengths and limitations

It must be remembered that group consensus, as achieved in this study, is not synonymous with best or correct practice (Trevelyan and Robinson, 2015). However, it was not the purpose of this research to gain consensus on the best approach; the purpose was to gain agreement on how an external focus can be implemented in clinical practice, if that is the chosen approach. As with all Delphi studies, there is also the challenge of what constitutes an “expert,” and on the ideal panel size. This study involved a small panel, who were purposively selected to represent a range of professional groups and expertise. While this gives diversity to group and strengthens overall opinion, the invitation list was not exhaustive and the views may not reflect those of a wider or different group. Therefore, we can only report agreement between the panelists that took part. This is justifiable given the scale and purpose of the research, keeping data generation manageable and focused.

The Delphi process was initiated with a set of pre-developed exercise examples. While this is acceptable given the purpose of this research, the existing research in this field, and the need to minimize burden on participants, it does mean that the opportunity for freely generated ideas may have been constrained.

Despite these limitations, the modified Delphi approach has many advantages. It enabled a range of experts, from different settings and a wide geographical spread, to inform the development of the next stage of this trial. Thus, it was a process toward achieving a wider aim, rather than providing a conclusive answer. Importantly, the use of Delphi methodology in the protocol development phase of this research programme will strengthen the overall study design, in line with published guidance for the development and testing of complex interventions (Craig et al., 2006).

## Conclusion

The complexity of instructional statements and their role in the motor learning process should not be underestimated. Although instructional methods are an integral part of physiotherapy, therapists may not always give due consideration to their impact on performance, learning, and recovery. Thus, the opportunity for optimizing motor learning and therefore functional recovery may not be fully realized. General principles for designing externally focused tasks in people with stroke have been generated through this Delphi survey, as well as specific externally focused exercises that will now be tested as part of a “real-world” clinical trial. The study design has considered implementation from the outset, in order to overcome barriers known to limit knowledge translation in stroke rehabilitation.

## Appendix. Examples of exercises with consensus for an external focus of attention

**Table:**

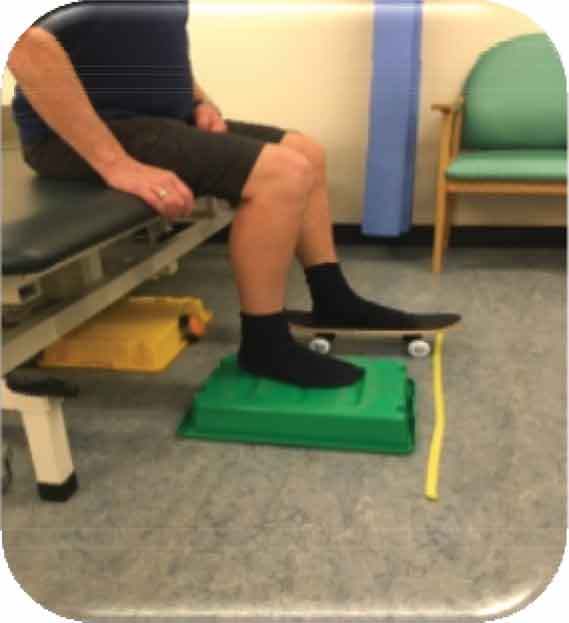
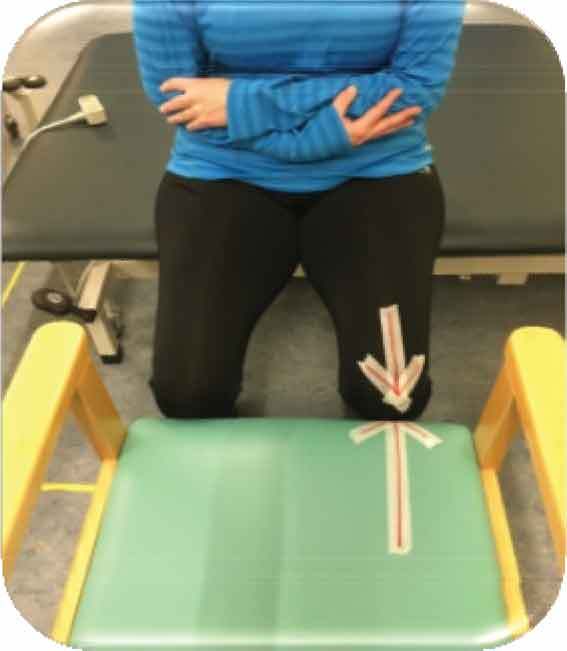

	
**Knee flexion in sitting** *“Hit the buzzer with the skateboard”*	**Hip control during sit to stand** *“Stand up, keeping the arrows pointing to each other”*

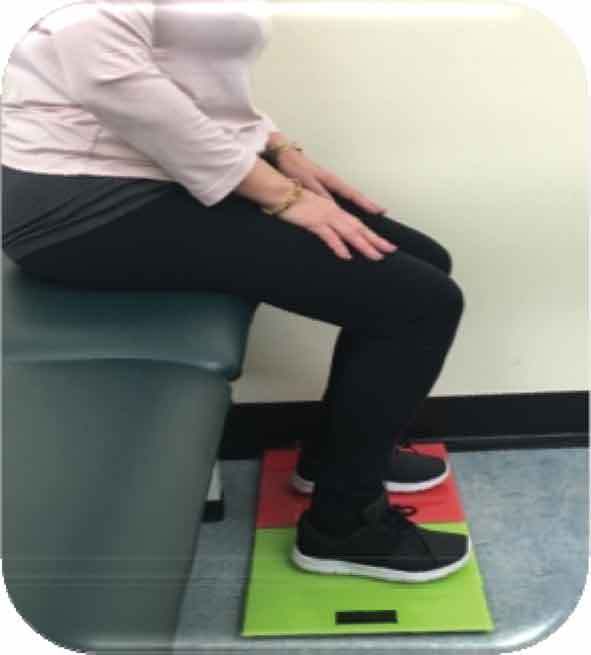
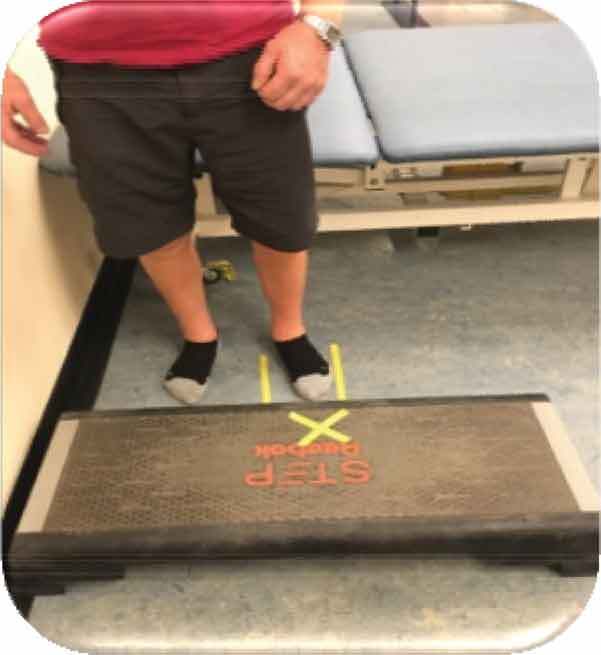
	
**Sit to stand, increasing weight transfer onto affected side** *“Push 20 kg through the red scales as you stand”*	**Step up onto a block** *“Touch the cross, and then back between the lines”*
Practicing left-knee flexion using a skateboard to reduce friction. Tape to mark position in front. Buzzer behind to give external focus of attention auditory feedback when adequate flexion is achieved. Partial sit to stand with arrow taped onto thigh, and onto surface in front. External focus of attention through aligning the arrows. Sit to stand with feet placed on electronic weighing scales. Therapist provides external focus of attention feedback by reporting the weight pushed through the scales. Step up onto a block. Tape to mark start/finish foot position on floor. Cross to provide external focus of attention target to step onto.	
